# Contribution of endogenous antibodies to learning deficits and astrocytosis in human P301S mutant tau transgenic mice

**DOI:** 10.1038/s41598-020-70845-x

**Published:** 2020-08-14

**Authors:** Julia van der Hoven, Annika van Hummel, Magdalena Przybyla, Prita R. Asih, Mehul Gajwani, Astrid F. Feiten, Yazi D. Ke, Arne Ittner, Janet van Eersel, Lars M. Ittner

**Affiliations:** 1 Dementia Research Centre and Department of Biomedical Sciences, Faculty of Medicine and Health Sciences, Macquarie University, Sydney, NSW 2109 Australia; 2 School of Medical Sciences, Faculty of Medicine, University of New South Wales, Sydney, NSW 2052 Australia

**Keywords:** Biological models, Animal disease models, Dementia, Alzheimer's disease, Experimental models of disease, Learning and memory, Cellular neuroscience, Alzheimer's disease, Dementia, Alzheimer's disease, Neuronal development, Neuroscience, Neuroimmunology, Autoimmunity, Autoimmune diseases, Immunology, Adaptive immunity, Humoral immunity, Antibodies

## Abstract

Antibodies have been explored extensively as a potential therapeutic for Alzheimer’s disease, where amyloid-β (Aβ) peptides and the tau protein deposit in patient brains. While the major focus of antibody-based therapy development was on Aβ, arguably with limited success in clinical trials, targeting tau has become an emerging strategy, possibly extending therapies to dementias with isolated tau pathology. Interestingly, low titres of autoantibodies to pathological tau have been described in humans and transgenic mouse models, but their pathophysiological relevance remained elusive. Here, we used two independent approaches to deplete the B-cell lineage and hence antibody formation in human P301S mutant tau transgenic mice, TAU58/2. TAU58/2 mice were either crossed with the B-cell-deficient *Ighm* knockout line (muMT^−/−^) or treated with anti-CD20 antibodies that target B-cell precursors. In both models, B-cell depletion significantly reduced astrocytosis in TAU58/2 mice. Only when B-cells were absent throughout life, in TAU58/2.muMT^−/−^ mice, were spatial learning deficits moderately aggravated while motor performance improved as compared to B-cell-competent TAU58/2 mice. This was associated with changes in brain region-specific tau solubility. No other relevant behavioural or neuropathological changes were observed in TAU58/2 mice in the absence of B-cells/antibodies. Taken together, our data suggests that the presence of antibodies throughout life contributes to astrocytosis in TAU58/2 mice and limits learning deficits, while other deficits and neuropathological changes appear to be independent of the presence of B-cells/antibodies.

## Introduction

Dementia, including its most common form, Alzheimer’s disease (AD), is one of the major causes of disability affecting older people, with an estimated 50 million people living with a form of dementia globally. Increased life expectancy is predicted to result in the greatest increase in prevalence in the next decade^1^. This is further amplified by unexplained increasing prevalence of dementia at ages between 65 and 80^2^. Together, this increases the urgency to understand underlying disease mechanisms and eventually develop effective treatments. Antibody-based therapeutic approaches have been extensively explored, although with limited success when targeting Aβ^3–5^. Therefore, current immunotherapeutic developments increasingly focus on alternative targets, in particular the neuronal microtubule-associated protein tau. While known causes of dementia have been identified as genetic mutations^6,7^ and traumatic brain injuries^8^, whether endogenous antibody (= autoantibody)-mediated processes contribute to disease onset and progression remains unknown.

The microtubule-associated protein tau is the main component of neurofibrillary tangle (NFT) pathology in AD and other dementias. Tau has over 80 predicted phosphorylation sites, many of which are phosphorylated in physiological processes (e.g. brain development^9^) and disease^10–12^. Phosphorylation of tau leads to changes in conformation, solubility and activity under both physiological and pathological conditions^13^. Pathogenic mutations in the tau-encoding *MAPT* gene underlying familial frontotemporal dementia (FTD) have assisted in the generation of multiple transgenic mouse models that recapitulate pathological and/or behavioural aspects of dementia^14–16^. This includes the TAU58/2 line more recently developed by us; TAU58/2 mice express human P301S mutant tau in neurons and present with progressive tau hyperphosphorylation and NFT pathology, as well as early-onset motor, behavioural and learning deficits^17^.

Using such transgenic mouse models, tau has been extensively targeted pre-clinically in both active and passive immunization approaches, showing efficacy in reducing tau pathology and in some studies improving cognitive and behavioural deficits^18,19^. Interestingly, both non-immunized tau transgenic mice and human AD/FTD patients showed baseline titres of autoantibodies to pathological tau^18,20^. Furthermore, autoantibodies to tau have been commonly found in the serum and cerebrospinal fluid of healthy adults as well as children^21,22^. The relevance of autoantibodies in the context of tau pathology is unknown.

Here, we crossed TAU58/2 mice with B-cell-deficient muMT^−/−^ mice or depleted B-cells in young TAU58/2 mice using anti-CD20 antibodies to explore the role of the adaptive immune system, specifically antibodies and attributable neuroinflammation, in the context of tau pathology. This augmented learning deficits of TAU58/2 mice while astrocytosis was reduced, suggesting a contribution of antibodies to disease onset and progression. This knowledge may improve understanding of the connection between antibodies and tau in disease.

## Results

### Genetic B-cell depletion from birth augmented learning deficits in tau transgenic mice

**Figure 1:**
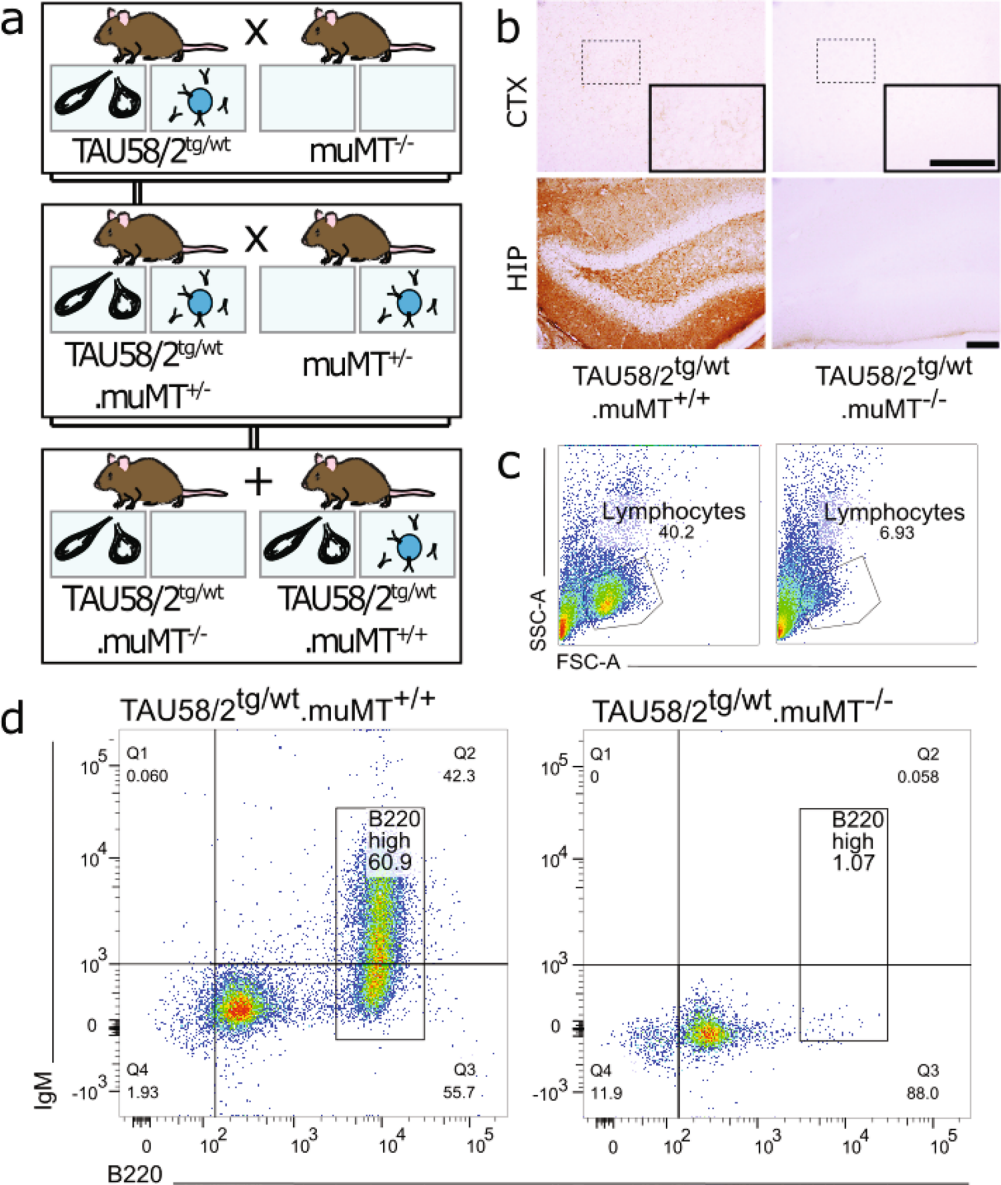
TAU58/2.muMT^−/−^ mice lack mature B-cells. (**a**) Breeding strategy: heterozygous TAU58/2 mice were crossed with muMT^−/−^ mice to obtain TAU58/2^tg/wt^.muMT^+/-^ offspring. These double heterozygous mice were then crossed with heterozygous muMT^+/-^ mice to produce the experimental cohort of mice that express the pathogenic human tau (depicted as black tangles), with or without B-cells and hence antibodies (as well as their non-transgenic tau littermates). (**b**) Endogenous antibody staining (brown) present in the cortex (top) and hippocampus (bottom) of TAU58/2^tg/wt^.muMT^+/+^ mice but absent in TAU58/2^tg/wt^.muMT^−/−^ mice. Inset, 2 × magnification of dashed outline in cortex. Scale bars, 100 µm. (**c**,**d**) FACS of splenic lymphocytes. (**c**) Gating of lymphocyte population. (**d**) Double positive B220^high^IgM^+^ lymphocytes present in TAU58/2^tg/wt^.muMT^+/+^ mice (left) but virtually completely absent in the TAU58/2^tg/wt^.muMT^−/−^ mice (right).

To determine a possible contribution of antibodies to phenotype and pathology of TAU58/2 mice, we first crossed them with muMT^−/−^ mice (Fig. 1a). muMT^−/−^ mice have a targeted disruption in transmembrane region of the *Ighm* gene encoding the heavy chain segment of IgM antibodies which are expressed on the surface membrane at the pre-B lymphocyte stage of cell development^23^. This membrane expression is required for progression from the pre-B lymphocyte stage to immature, mature and antibody-secreting plasma cell stages^24^. Therefore, muMT^−/−^ homozygous mice are unable to produce B lymphocytes and antibodies. First, we confirmed the absence of antibodies and B-cells in the resulting TAU58/2.muMT^−/−^ mice compared to TAU58/2.muMT^+/+^ littermates. Staining of brain sections with antibodies to mouse IgG showed virtually no labelling in TAU58/2.muMT^−/−^ brains, compared with intense staining of TAU58/2.muMT^+/+^ tissue (Fig. 1b). Fluorescence-activated cell sorting (FACS) of spleens from TAU58/2.muMT^−/−^ confirmed a lack of IgM^+^/B220^high^ lymphocytes, suggesting arrest of B lymphocyte development at the pre-B lymphocyte phase in these mice (Fig. 1c,d). Taken together, TAU58/2.muMT^−/−^ mice lacked endogenous antibodies and B lymphocytes.

**Figure 2:**
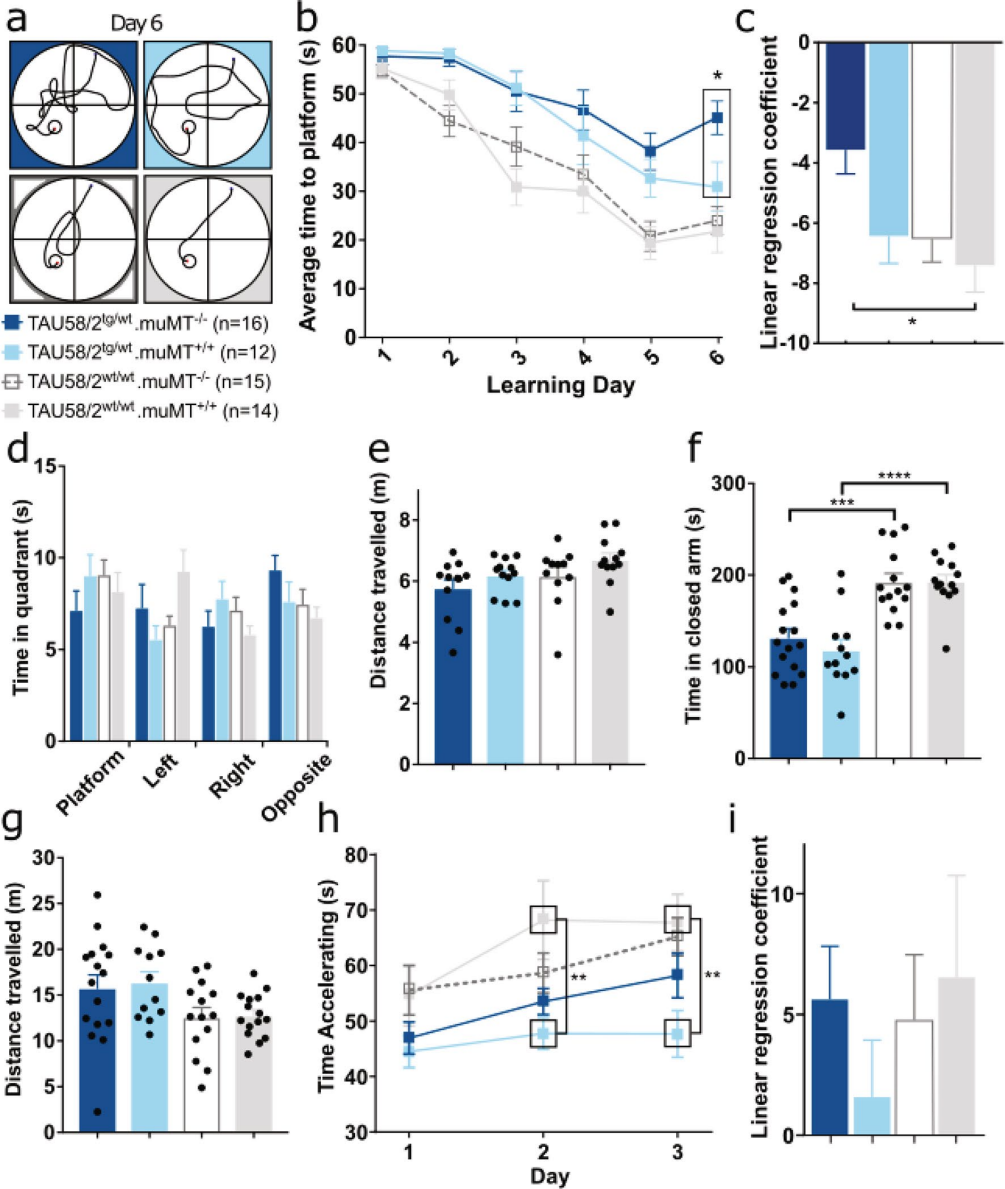
Augmented learning deficits and improved motor performance in TAU58/2.muMT^−/−^ mice. (**a**) Representative swim paths on day 6 for all tested genotypes (*n*-numbers of mice tested per group). (**b**) During the learning phase of the MWM, TAU58/2^tg/wt^.muMT^−/−^ mice showed a significantly increased latency to find the platform on day 6 (*P = 0.029 vs. TAU58/2^tg/wt^.muMT^+/+^). (**c**) Linear regression of escape latency curves confirmed slower learning of TAU58/2^tg/wt^.muMT^−/−^ mice compared with TAU58/2^wt/wt^.muMT^+/+^ littermates over 6 test days (*P = 0.0115). (**d**) In MWM probe trials, TAU58/2^tg/wt^.muMT^−/−^ mice spent less time in the platform quadrant and more time in the opposite quadrant than all other genotypes. (**e**) No significant differences in the average distance swum between experimental groups. (**f**) Both TAU58/2^tg/wt^.muMT^−/−^ and TAU58/2^tg/wt^.muMT^+/+^ mice showed similar reduced times in closed arms during EPM testing as compared to TAU58/2^wt/wt^.muMT^−/−^ and TAU58/2^wt/wt^.muMT^+/+^ littermates, respectively (***P = 0.002, ****P < 0.0001). (**g**) Distance travelled in the EPM was similar for all genotypes. (**h**) Latency to fall remained significantly decreased on days 2 and 3 of Rotarod testing in TAU58/2^tg/wt^.muMT^+/+^ mice, while TAU58/2^tg/wt^.muMT^−/−^ mice improved similarly to TAU58/2^wt/wt^.muMT^−/−^ and TAU58/2^wt/wt^.muMT^+/+^ littermates (**P = 0.008 and 0.009). (**i**) Linear regression of fall latencies confirmed improved performance of TAU58/2^tg/wt^.muMT^−/−^ mice over consecutive testing days, while TAU58/2^tg/wt^.muMT^+/+^ littermates failed to improve. All graphs are displayed as group means ± S.E.M. (error bars).

We have determined that mature TAU58/2 mice show delayed spatial learning in the Morris water maze (MWM) (Przybyla M et al*.*, unpublished). To assess possible effects of the lack of antibodies on spatial learning, 8-month-old TAU58/2.muMT^−/−^ and TAU58/2.muMT^+/+^ mice and non-transgenic muMT^−/−^ and muMT^+/+^ littermates were subjected to the MWM paradigm. TAU58/2.muMT^−/−^ mice showed significantly delayed learning after 6 days of MWM testing when compared with TAU58/2.muMT^+/+^ littermates (Fig. 2a–c). Both non-transgenic muMT^−/−^ and muMT^+/+^ controls showed similarly accelerated learning compared to TAU58/2.muMT^+/+^ mice, consistent with intact spatial learning. Probe trials after removal of the hidden escape platform showed a trend towards reduced time TAU58/2.muMT^−/−^ mice spent in the target quadrant and increased time in the opposite quadrant of the MWM as compared with controls, but differences did not reach significance (Fig. 2d). There was no overt difference in the average distance swum between groups (Fig. 2e). Taken together, TAU58/2.muMT^−/−^ mice presented with delayed learning in the MWM task.

TAU58/2 mice present with early-onset disinhibition and increased open arm time in the elevated plus maze (EPM) paradigm^25^. Therefore, we tested TAU58/2.muMT^−/−^ and TAU58/2.muMT^+/+^ mice and non-transgenic muMT^−/−^ and muMT^+/+^ littermates in the EPM. TAU58/2.muMT^−/−^ mice spent less time in the closed arms of the EPM as did their TAU58/2.muMT^+/+^ littermates relative to non-transgenic muMT^−/−^ and muMT^+/+^ mice (Fig. 2f). However, no difference in time spent in the closed arms was observed between the TAU58/2.muMT^−/−^ and TAU58/2.muMT^+/+^ mice. Movement of mice during EPM testing was comparable between all groups (Fig. 2g). Together, these findings indicate no obvious effects of the lack of antibodies on the TAU58/2 disinhibition phenotype.

Finally, we tested the effects of antibody-depletion on motor deficits of TAU58/2 mice^17^. While both TAU58/2.muMT^−/−^ and TAU58/2.muMT^+/+^ mice presented with reduced performance during motor testing on the accelerating Rotarod as compared with non-transgenic muMT^−/−^ and muMT^+/+^ mice on day 1, TAU58/2.muMT^+/+^ continued to perform significantly worse compared with non-transgenic littermates on days 2 and 3 (Fig. 2h). Interestingly, TAU58/2.muMT^−/−^ mice improved their performance on the Rotarod over the 3 test days, while TAU58/2.muMT^+/+^ mice showed no improvements (Fig. 2h,i). Taken together, lack of antibodies did not significantly improve motor deficits of TAU58/2 mice but enabled progressive learning of the task during subsequent test days.

### Genetic B-cell depletion from birth reduced astrocytosis in tau transgenic mice

**Figure 3:**
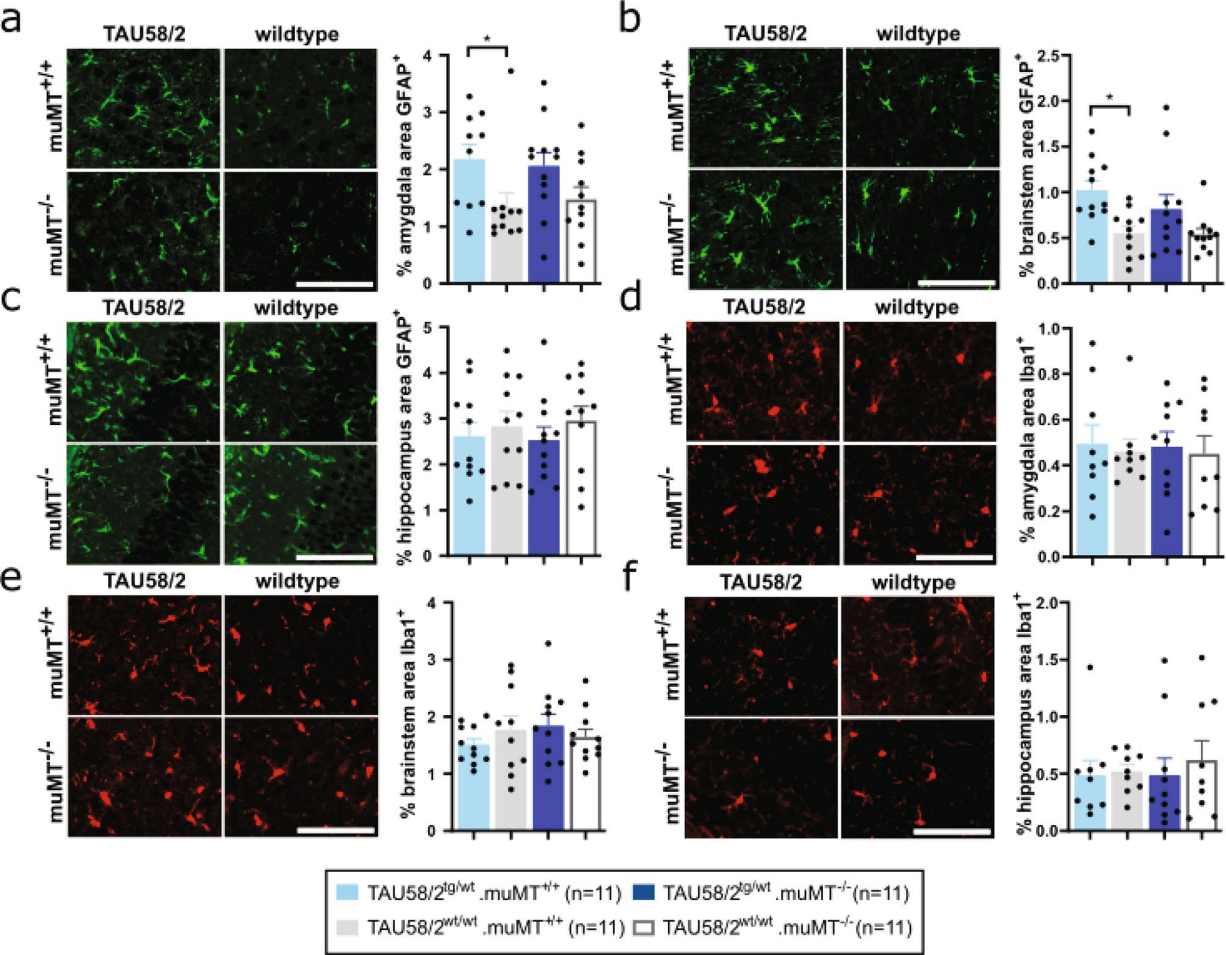
Reduced astrogliosis in TAU58/2.muMT^−/−^ mice. (**a**) Representative GFAP staining of astrocytes (green) and quantification showing increased astrocytosis in the amygdala of TAU58/2^tg/wt^.muMT^+/+^ mice compared with TAU58/2^wt/wt^.muMT^+/+^ littermates, while staining was comparable in TAU58/2^tg/wt^.muMT^−/−^ mice and TAU58/2^wt/wt^.muMT^−/−^ controls (*P = 0.0319). (**b**) Increased astrocytosis in the brainstem of TAU58/2^tg/wt^.muMT^+/+^ mice compared with TAU58/2^wt/wt^.muMT^+/+^ littermates, while staining was comparable in TAU58/2^tg/wt^.muMT^−/−^ mice and TAU58/2^wt/wt^.muMT^−/−^ controls (*P = 0.019). (**c**) GFAP staining of hippocampus was comparable for all genotypes. (**d**–**f**) Representative Iba1 staining of microglia and quantification showed no overt difference in (**d**) amygdala, (**e**) brainstem and (**f**) hippocampus between genotypes. Legend and sample numbers below figures. Scale bar = 100 µm for all images.

We have previously reported astrocytosis and microgliosis associated with NFT pathology in TAU58/2 mice^17^. To investigate whether lack of antibodies influences inflammation in the TAU58/2.muMT^−/−^ mice, we assessed astrocytes and microglia in the amygdala and hippocampus by staining brain sections with the respective cell-type markers GFAP and Iba1. Consistent with our previous report^17^, 8-month-old TAU58/2.muMT^+/+^ mice presented with significant astrocytosis in the amygdala and brainstem when compared to non-transgenic muMT^+/+^ mice (Fig. 3a,b). In contrast, there was no significantly increased labelling of GFAP^+^ astrocytes in TAU58/2.muMT^−/−^ mice as compared with non-transgenic muMT^−/−^ and muMT^+/+^ mice, suggesting reduced astrocytosis in the absence of B-cells and antibodies. GFAP-staining was not increased in the hippocampus of TAU58/2.muMT^+/+^ brains (Fig. 3c). Accordingly, there was no significant difference in astrocyte labelling between the groups, although GFAP staining showed a trend towards reduction in TAU58/2.muMT^−/−^ mice as compared with the other groups. Labelling of A1 (= neurotoxic) and A2 (= neuroprotective) astrocyte subpopulations with antibodies to C3 and S100A10^25^, respectively, did not show subtype specific changes to astrocyte numbers upon genetic B-cell depletion in TAU58/2.muMT^−/−^ compared to TAU58/2.muMT^+/+^ amygdala, hippocampus and brainstem (Supplementary Fig. 1). In contrast to astrocytes, there were no changes to microglial numbers in the amygdala, hippocampus or brainstem of TAU58/2.muMT^−/−^ compared to TAU58/2.muMT^+/+^ or non-transgenic muMT^−/−^ and muMT^+/+^ controls (Fig. 3d–f). Similarly, there were no changes observed in microglial appearance or engulfment of NFT-like lesions in the amygdala and hippocampus of TAU58/2.muMT^−/−^ compared to TAU58/2.muMT^+/+^ mice (Supplementary Fig. 2). Taken together, absence of antibodies and B-cells reduced astroglial activation in TAU58/2 mice but did not alter the microglial response.

### Genetic B-cell depletion from birth did not substantially modulate tau pathology but affected its solubility in tau transgenic mice

**Figure 4:**
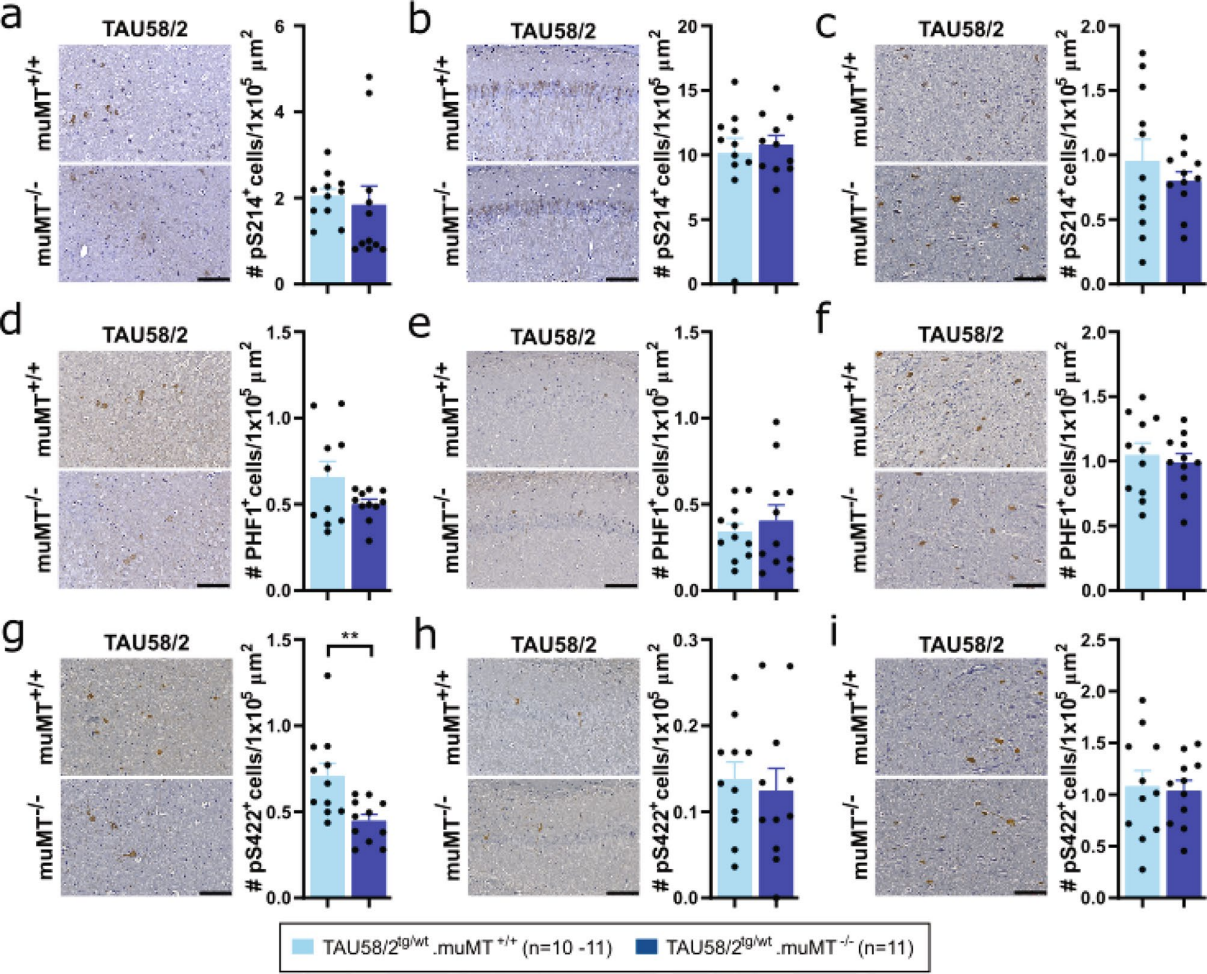
Tau pathology in TAU58/2.muMT^−/−^ mice. Representative images and quantification of immunohistochemistry with phosphorylation-site specific antibodies for (**a**–**c**) Ser214 (pS214), (**d**–**f**) Ser396/Ser404 (PHF1) and (**g**–**i**) Ser422 (pS422) of the (**a**,**d**,**g**) amygdala, (**b**,**e**,**h**) hippocampus and (**c**,**f**,**i**) brainstem from TAU58/2^tg/wt^.muMT^−/−^ mice compared with TAU58/2^tg/wt^.muMT^+/+^ littermates revealed no differences except for (**g**) pS422 in the amygdala (**P = 0.0056). Legend and sample numbers below figures. Scale bar = 100 µm for all images.

**Figure 5:**
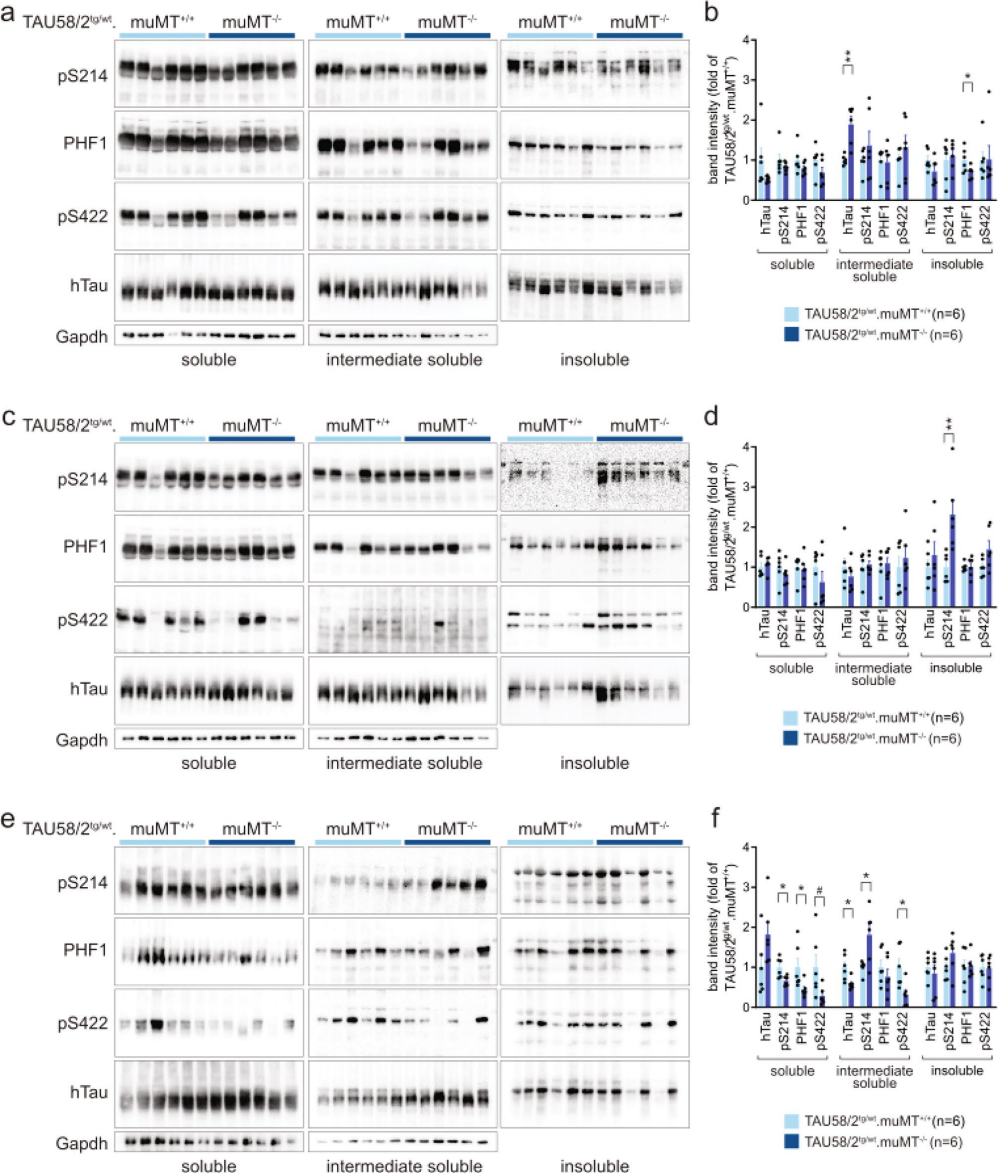
Altered tau solubility in TAU58/2.muMT^−/−^ mice. (**a**) Western blots of soluble, intermediate soluble and insoluble fractions from sequentially extracted hippocampus from TAU58/2^tg/wt^.muMT^−/−^ mice (n = 6) and TAU58/2^tg/wt^.muMT^+/+^ controls (n = 6). Blots were tested with phosphorylation-site specific antibodies for Ser214 (pS214), Ser396/Ser404 (PHF1) and Ser422 (pS422) and human tau (hTau). Loading of soluble and intermediate soluble fractions were controlled by detecting Gapdh. (**b**) Quantification of blots shown in (a) (*P = 0.047; **P = 0.0012). (**c**) Western blots of sequential extracts from cortex samples. (**d**) Quantification of blots shown in (c) (**P = 0.0083). (**e**) Western blots of sequential extracts from brainstem samples. (**f**) Quantification of blots shown in (e) (soluble: pS214: *P = 0.0401, PHF1: *P = 0.0132, pS422: ^#^P = 0.0540; intermediate soluble: hTau: *P = 0.0159, pS214: *P = 0.0150; pS422: *P = 0.0177). Full size blots are shown in Supplementary Fig. 4.

Tau becomes increasingly phosphorylated over time in TAU58/2 brains, eventually leading to NFT-like pathology^17^. Therefore, we examined the effects of the lack of antibodies on the phosphorylation of tau at different sites, using phosphorylation site-specific antibodies. Specifically, we stained brain sections of TAU58/2.muMT^−/−^ and TAU58/2.muMT^+/+^ mice with antibodies to the phosphorylated sites serine (S) 214 (pS214), S396 + S404 (PHF1) and S422 (pS422). Phosphorylation of the early pathological marker pS214 and late pathological markers PHF1 and pS422 were unchanged in the amygdala, hippocampus and brainstem of TAU58/2.muMT^−/−^ compared to TAU58/2.muMT^+/+^ brains (Fig. 4a–i). Notably, pS422 was significantly reduced in the amygdala of TAU58/2.muMT^−/−^ compared to TAU58/2.muMT^+/+^ mice. To determine whether genetic B-cell depletion impacted on solubility of tau in TAU58/2.muMT^−/−^ mice as compared to TAU58/2.muMT^+/+^ controls, we extracted hippocampal, cortical and brainstem brain tissue individually by sequential extraction with buffers of increasing stringency. This revealed brain region-specific changes to tau solubility; In the hippocampus, levels of soluble human tau were lower, while they were significantly increased in the intermediate soluble fraction, without overt changes to insoluble tau in TAU58/2.muMT^−/−^ mice as compared to TAU58/2.muMT^+/+^ controls (Fig. 5a,b). Tau phosphorylation remained unchanged across fractions, with exception of significantly reduced PHF1 levels in the insoluble fraction of TAU58/2.muMT^−/−^ mice as compared to TAU58/2.muMT^+/+^ controls. Solubility of tau remained unchanged in cortical extracts between in TAU58/2.muMT^−/−^ and TAU58/2.muMT^+/+^ mice (Fig. 5c,d). However, phosphorylation of S214 was significantly increased in insoluble fractions of TAU58/2.muMT^−/−^ mice as compared to TAU58/2.muMT^+/+^ controls. Conversely to the hippocampus, soluble tau levels were increased and intermediate soluble tau levels significantly reduced in brainstem extracts of TAU58/2.muMT^−/−^ mice as compared to TAU58/2.muMT^+/+^ controls (Fig. 5e,f). While overall phosphorylation of tau in the soluble and intermediate soluble fractions were significantly reduced, S214 phosphorylation was significantly increased in the intermediate soluble fractions of TAU58/2.muMT^−/−^ mice as compared to TAU58/2.muMT^+/+^ controls. Insoluble tau levels and phosphorylation remained unchanged in brainstem extracts. Taken together, the absence of antibodies and B-cell did not fundamentally change the phosphorylation state of tau but had brain region-specific effects on its solubility in TAU58/2 mice.

### B-cell depletion in mature tau transgenic mice did not alter functional deficits

**Figure 6:**
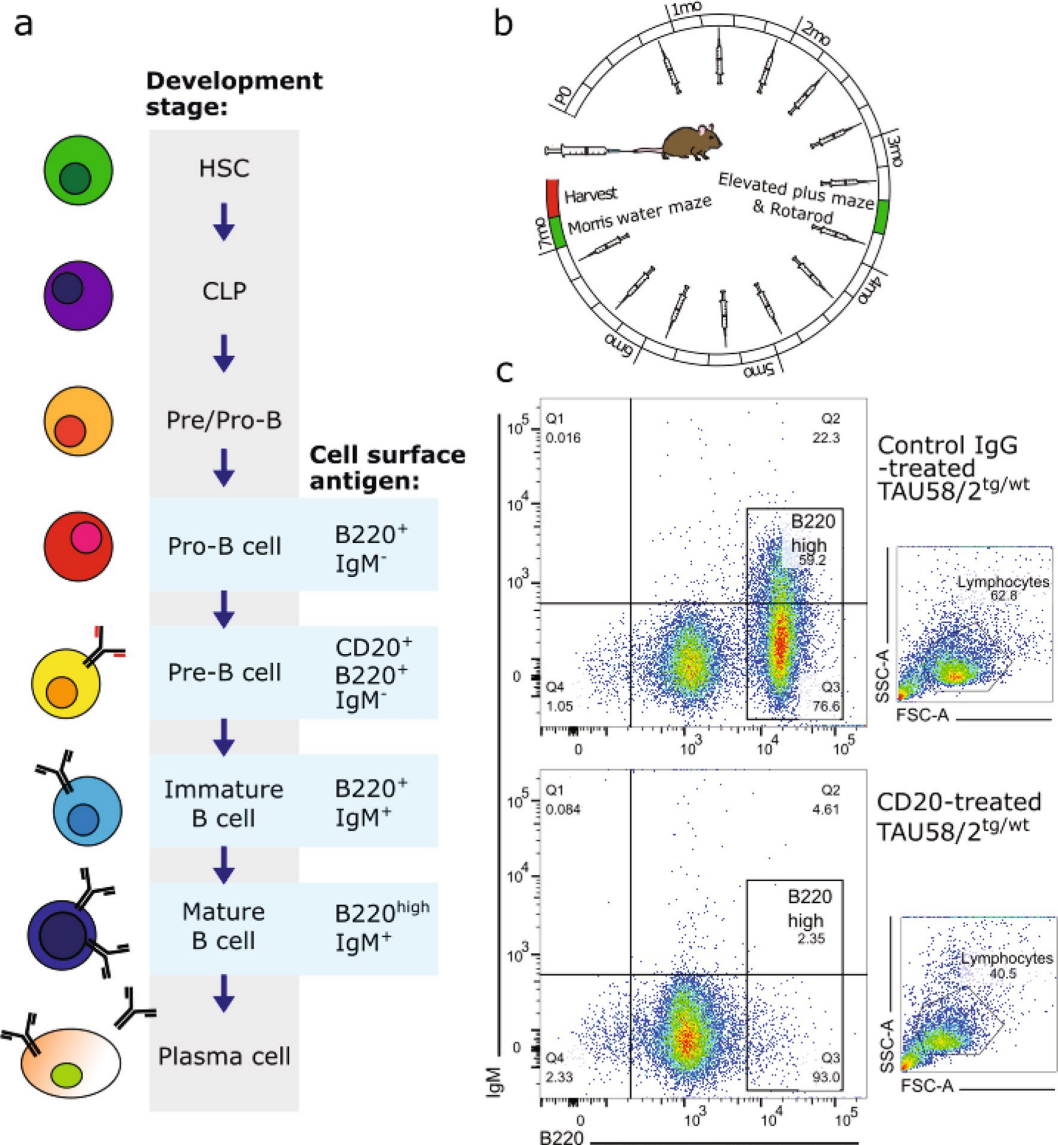
Antibody-mediated B-cell depletion in TAU58/2 mice. (**a**) Schematic of B-cell development from hematopoietic stem cell (HSC), common lymphoid progenitor (CLP), B220-positive pro-B-cells, CD20-positive pre-B cell to immature and mature IgM-positive B-cells. (**b**) B-cell depletion study design: From weaning at 21 days of age, TAU58/2 mice and their non-transgenic littermates received either anti-CD20 or control antibody via tail vein injection every two weeks to maintain B cell-depleted status. At 3.5 months of age, all mice were tested for disinhibition (EPM) and motor (Rotarod) phenotypes. Following the final injection and assessment of visuospatial memory and learning (MWM), brains were harvested for analysis. (**c**) FACS of splenic lymphocytes: Double positive B220^high^IgM^+^ lymphocytes were present in control IgG-treated TAU58/2^tg/wt^ mice (top) but virtually absent in the anti-CD20-treated TAU58/2^tg/wt^ mice (bottom).

Using a complementary model of B-cell depletion to the muMT^−/−^ line and in order to distinguish effects of B-cell depletion during development and maturation of the mice (= in utero to post-partum day 21) from later impacts, we treated TAU58/2 mice and their non-transgenic littermates with anti-CD20 (or control) antibodies. Anti-CD20 antibody treatment confers removal of mature B lymphocytes by targeting the CD20^+^ pre-B lymphocyte stage of plasma cell maturation (Fig. 6a). Depletion of B-cells was initiated at 3 weeks of age and thereafter maintained through fortnightly i.v. injections of anti-CD20 antibodies until 7 months of age (Fig. 6b). Control TAU58/2 and non-transgenic mice were treated with a control antibody using the same schedule. FACS analysis of spleens from TAU58/2 mice confirmed depletion of mature IgM^+^/B220^high^ B cells upon anti-CD20 treatment as compared to control treated mice (Fig. 6c).

**Figure 7:**
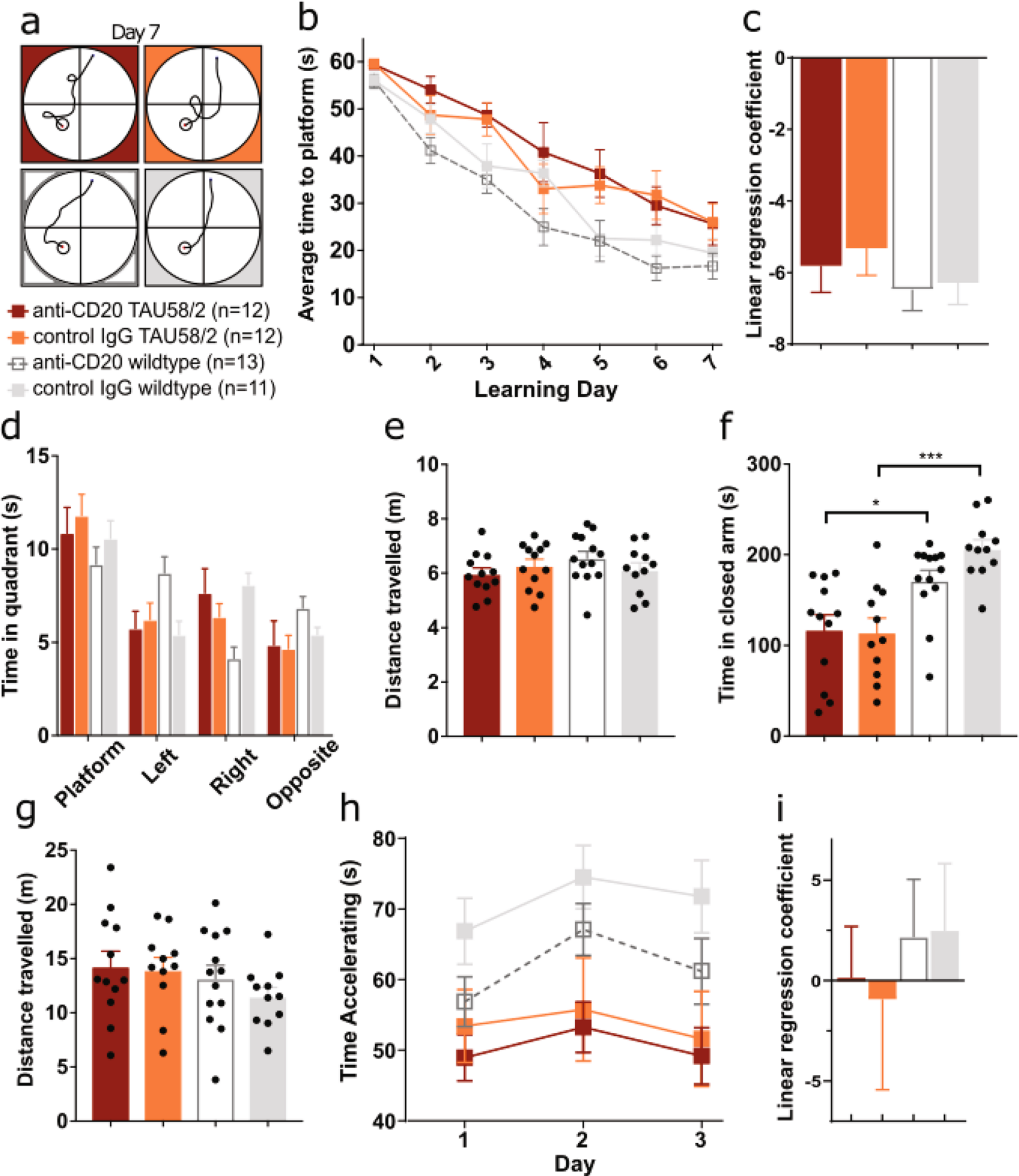
Antibody-mediated B-cell depletion did not change the functional deficits of TAU58/2 mice. (**a**) Representative swim paths on day 7 for all tested genotypes (*n*-numbers of mice tested per group). (**b**) Latency to find the platform was generally delayed in both TAU58/2 treatment cohorts compared with non-transgenic littermates but improved similarly over the 7 days in anti-CD20- and control IgG-treated TAU58/2 mice during the learning phase of the MWM. (**c**) Linear regression of escape latency curves confirmed comparable learning of anti-CD20- and control IgG-treated TAU58/2 mice over 7 test days. (**d**) In MWM probe trials, all genotypes spent equally more time in the platform quadrant. (**e**) No significant differences were observed in the average distance swum between experimental groups. (**f**) Both anti-CD20- and control IgG-treated TAU58/2 mice showed similar reduced times in closed arms during EPM testing as compared to treated non-transgenic littermates. (**g**) Distance travelled in the EPM was similar for all test groups. (**h**) Latency to fall remained significantly decreased after 3 days of Rotarod testing in anti-CD20- and control IgG-treated TAU58/2 mice as compared with treated non-transgenic littermates. (**i**) Linear regression of fall latencies confirmed no improvements in anti-CD20- and control IgG-treated TAU58/2 mice. All graphs are displayed as group means ± S.E.M. (error bars).

In contrast to TAU58/2.muMT^−/−^ mice, anti CD20-treated TAU58/2 mice showed no changes to the spatial learning deficits observed in control IgG-treated TAU58/2 mice when compared to anti CD20- or control IgG-treated non-transgenic littermates (Fig. 7a–c). Similarly, anti CD20- and control IgG-treated TAU58/2 mice spent comparable times in the target quadrant (Fig. 7d). There was no overt difference in average distance swum between groups (Fig. 7e). Both anti-CD20- and control IgG-treated TAU58/2 mice showed similar disinhibition behaviour during EPM testing with reduced closed arm time as compared to anti-CD20- and control IgG-treated non-transgenic mice (Fig. 7f). Movement of mice during EPM testing was comparable between all groups (Fig. 7g). Finally, anti-CD20- and control IgG-treated TAU58/2 mice showed similar deficits during Rotarod testing without improvement over consecutive test days (Fig. 7h,i). Taken together, long-term B-cell depletion in mature TAU58/2 mice did not alter learning, behavioural or motor deficits.

### B-cell depletion in mature tau transgenic mice reduced astrocytosis

**Figure 8:**
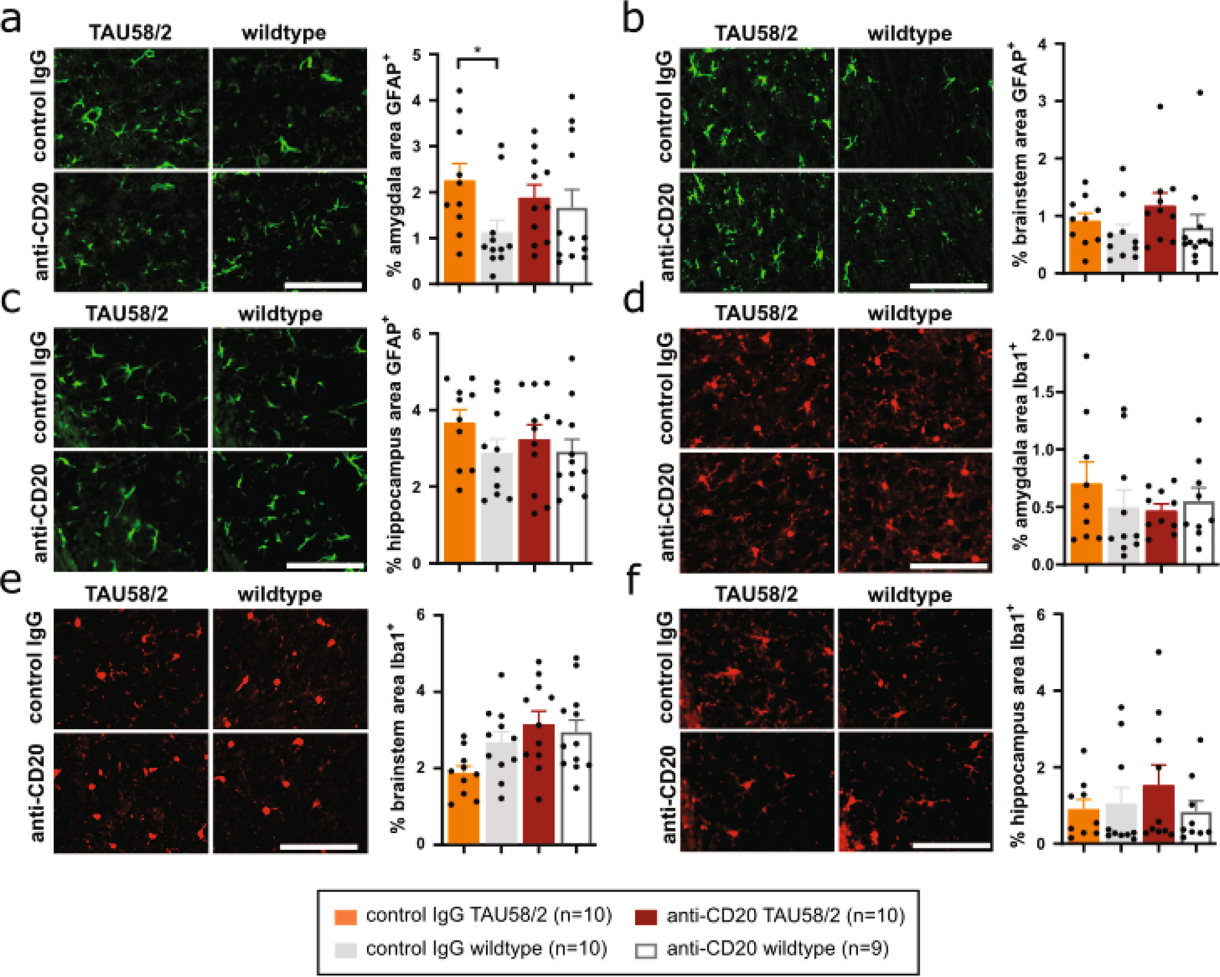
Reduced astrogliosis upon antibody-mediated B-cell depletion in TAU58/2 mice. (**a**) Representative GFAP staining of astrocytes (green) and quantification showing increased astrocytosis in the amygdala of control IgG-treated TAU58/2 mice compared with IgG-treated wild type littermates, while staining was comparable in anti-CD20-treated TAU58/2 and wildtype littermates (*P = 0.0225). (**b**,**c**) GFAP staining of (**b**) brainstem and (**c**) hippocampus were comparable for all experimental groups. (**d**–**f**) Representative Iba1 staining of microglia and quantification showed no overt difference in the (**d**) amygdala, (**e**) brainstem and (**f**) hippocampus between test groups. Legend and sample numbers below figures. Scale bar = 100 µm for all images.

When assessing neuropathological changes after long-term B-cell depletion in mature TAU58/2 mice, we found that, similar to TAU58/2.muMT^−/−^ mice, anti-CD20-treated TAU58/2 mice presented with comparable labelling of GFAP^+^ astrocytes in the amygdala as in anti-CD20-treated non-transgenic littermates, while control IgG-treated TAU58/2 mice showed significant astrocytosis as compared with control IgG-treated non-transgenic mice (Fig. 8a). GFAP-staining was not increased in the brainstem or hippocampus of anti-CD20- or control IgG-treated TAU58/2 mice compared to non-transgenic controls (Fig. 8b,c). Labelling of A1 and A2 astrocyte subpopulations with antibodies to C3 and S100A10^25^, respectively, did not show subtype specific changes to astrocyte numbers upon genetic B-cell depletion in anti-CD20-treated TAU58/2 compared to control amygdala, hippocampus and brainstem (Supplementary Fig. 1). In contrast to astrocytes, there were no changes to microglial numbers in the amygdala or hippocampus of anti-CD20- or control IgG-treated TAU58/2 mice as compared to the accordingly treated non-transgenic controls (Fig. 8d–f). There was no change in microglial appearance or engulfment of NFT-like lesions, as described earlier by us for the TAU58/2 line^17^, in the amygdala and brainstem of anti-CD20-treated TAU58/2 mice (Supplementary Fig. 3). Interestingly, the number of microglia closely associated with NFT-like lesions were reduced in the hippocampus of anti-CD20-treated TAU58/2 mice compared with TAU58/2 mice that received control IgG injections (Supplementary Fig. 3b). Taken together, absence of antibodies induced by long-term B-cell depletion in mature mice reduced astroglial activation in TAU58/2 mice and altered microglial behaviour.

### Altered tau pathology upon B-cell depletion in mature tau transgenic mice

**Figure 9:**
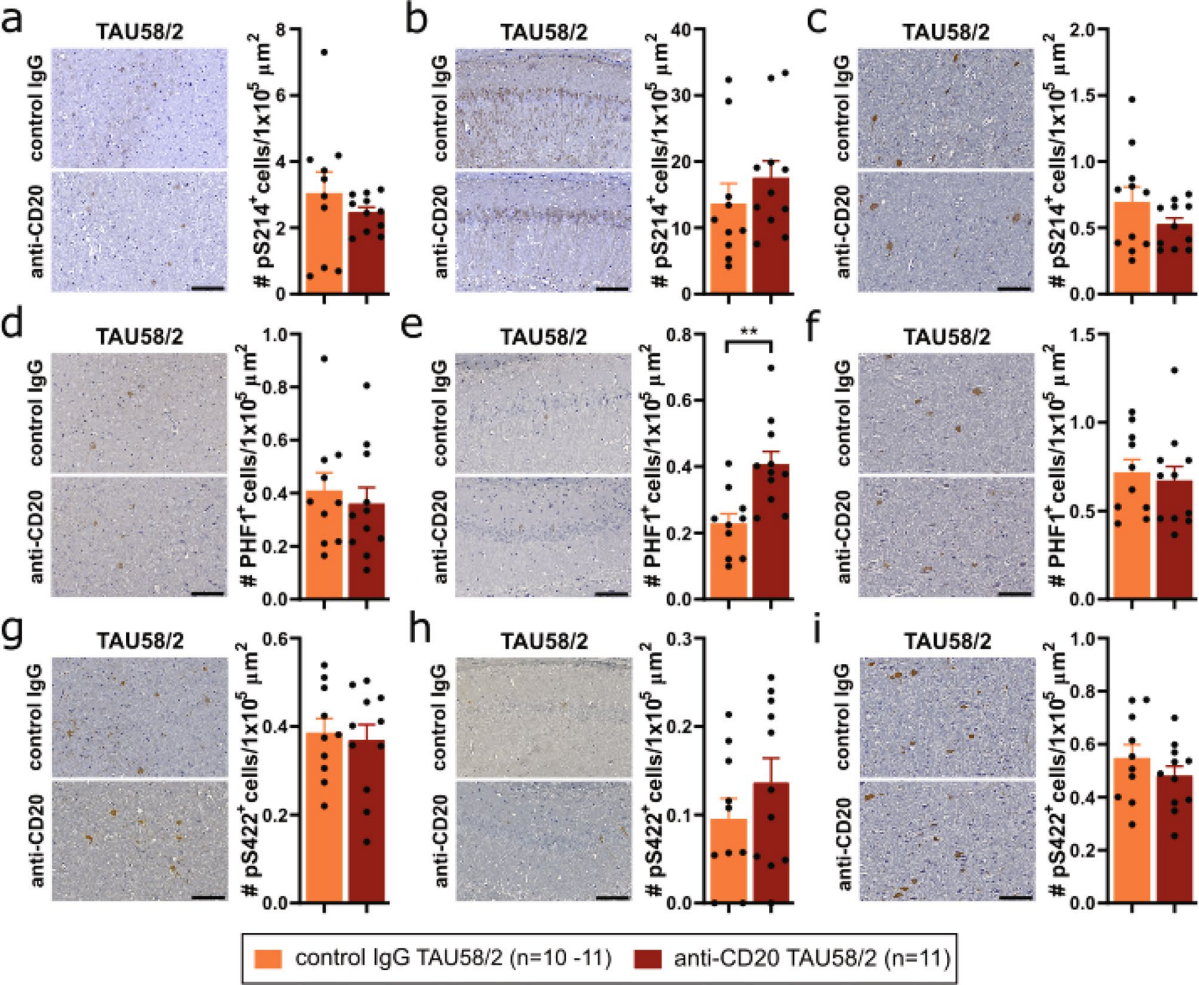
Tau pathology upon antibody-mediated B-cell depletion in TAU58/2 mice. Representative images and quantification of immunohistochemistry with phosphorylation-site specific antibodies for (a-c) Ser214 (pS214), (**d**–**f**) Ser396/Ser404 (PHF1) and (**g**–**i**) Ser422 (pS422) of the (**a**,**d**,**g**) amygdala, (**b**,**e**,**h**) hippocampus and (**c**,**f**,**i**) brainstem from anti-CD20 and control IgG-treated TAU58/2 mice revealed no differences except for (**e**) PHF1 in the hippocampus (**P = 0.0026). Legend and sample numbers below figures. Scale bar = 100 µm for all images.

**Figure 10:**
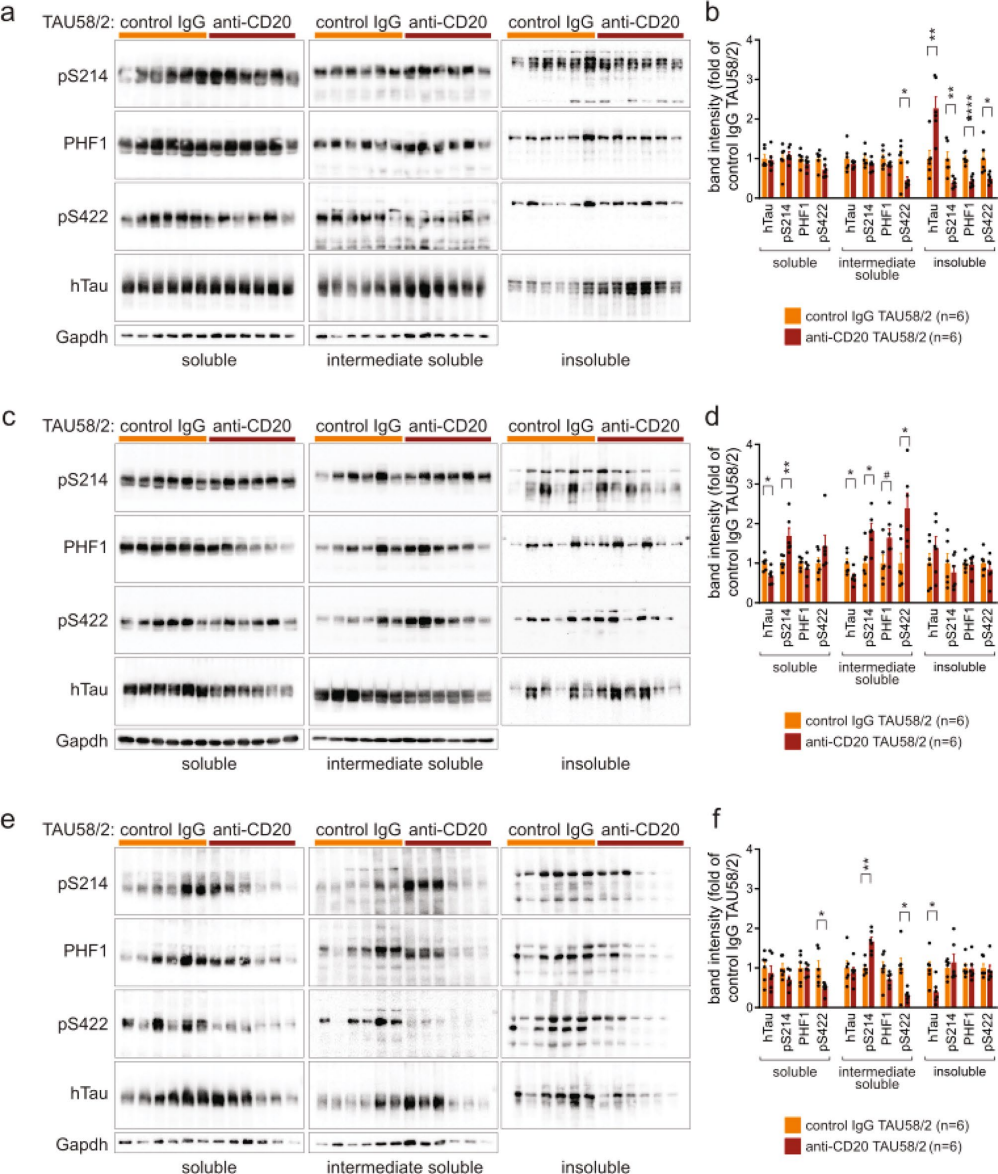
Altered tau solubility upon antibody-mediated B-cell depletion in TAU58/2 mice. (**a**) Western blots of soluble, intermediate soluble and insoluble fractions from sequentially extracted hippocampus from TAU58/2^tg/wt^.muMT^−/−^ mice (n = 6) and TAU58/2^tg/wt^.muMT^+/+^ controls (n = 6). Blots were tested with phosphorylation-site specific antibodies for Ser214 (pS214), Ser396/Ser404 (PHF1) and Ser422 (pS422) and human tau (hTau). Loading of soluble and intermediate soluble fractions were controlled by detecting Gapdh. (**b**) Quantification of blots shown in (**a**) (intermediate soluble: pS422: *P = 0.0295; insoluble: hTau: **P = 0.0048, pS214: **P = 0.0056, PHF1: ****P = 0.00003, pS422: *P = 0.0133). (**c**) Western blots of sequential extracts from cortex samples. (**d**) Quantification of blots shown in (**c**) (soluble: hTau: *P = 0.0135, pS214: **P = 0.0075; intermediate soluble: hTau: *P = 0.0260, pS214: *P = 0.0112, PHF1: ^#^P = 0.0634, pS422: *P = 0.0134). (**e**) Western blots of sequential extracts from brainstem samples. (**f**) Quantification of blots shown in (e) (soluble: pS422: *P = 0.0224; intermediate soluble: pS214: **P = 0.0053; pS422: *P = 0.0201; insoluble: hTau: *P = 0.0122). Full size blots are shown in Supplementary Fig. 5.

Similar to TAU58/2 crossed with muMT^−/−^ mice, we stained brain sections of anti-CD20- and control IgG-treated TAU58/2 mice with antibodies to the phosphorylated sites S214 (pS214), S396 + S404 (PHF1) and S422 (pS422). Except for increased PHF1 phosphorylation in the hippocampus, phosphorylation of tau at all these sites were not significantly changed in either the amygdala, hippocampus or brainstem of anti-CD20-treated TAU58/2 mice as compared to control IgG-treated TAU58/2 mice (Fig. 9a–i). When assessing tau solubility biochemically, we found significantly increased levels of insoluble tau in the hippocampus of anti-CD20-treated TAU58/2 mice as compared to controls, with concomitant significant reduction of tau phosphorylation at all tested sited in the insoluble fractions (Fig. 10a,b). Only S422 phosphorylation was significantly reduced in the soluble and intermediate soluble fractions, with other sites and tau levels remaining similar in anti-CD20-treated TAU58/2 hippocampus as compared with controls. For comparison, soluble and insoluble soluble tau levels were significantly reduced, while levels of tau phosphorylation at S214 in soluble fractions and all sites in the intermediate soluble fractions of cortical tissue were significantly increased in anti-CD20-treated TAU58/2 mice as compared to controls (Fig. 10c,d). In brainstem extracts, insoluble tau levels were significantly lower in anti-CD20-treated TAU58/2 mice as compared to controls, with unchanged phosphorylation (Fig. 10e,f). Phosphorylation of tau at S214 was significantly increased in soluble and at S422 significantly decreased in soluble and intermediate soluble fractions. Taken together, absence of antibodies due to long-term depletion of B-cells in mature mice did not change the histopathological patterns of tau phosphorylation but had brain regions-specific effects on tau solubility in TAU58/2 mice.

## Discussion

In the present study, we show that absence of antibodies and B-cells reduced astrocytosis in the brains of P301S tau transgenic TAU58/2 mice. Experimental reduction of immunoglobulins and plasma cells was achieved either by genetically depleting B-cells throughout the lifetime of the mice or by long-term depletion after mice had fully developed. In contrast, only lifetime depletion of B-cells modulated learning deficits in TAU58/2, with further augmented acquisition deficits during MWM testing but improved motor performance in TAU58/2.muMT^−/−^ mice.

TAU58/2 mice present with a variety of motor and behavioural deficits, including progressive spatial learning deficits, similar to other tau transgenic mouse models of FTD and AD^17,26,27^. Comprehensive testing of TAU58/2 mice crossed with the muMT^−/−^ line or after long-term anti-CD20 antibody-mediated B-cell depletion showed that antibodies (or other related B-cell mediated effects) play no major role in functional deficits associated with transgenic expression of mutant human tau and tau pathology. However, the very distinct effect of genetic B-cell depletion on the spatial and motor learning deficits of TAU58/2 mice may suggest that pathological pathways that involve antibodies are limited to distinct neuronal populations or networks during the early onset of deficits. Hence, later B-cell depletion has no impact on this phenotype. An alternative explanation that cannot be fully excluded is that the genetic modification itself causes effects due to unknown neuronal functions of *Ighm* gene products. Such effects would however be surprisingly specific for learning, since both genetic and anti-CD20 antibody-mediated B-cell depletion have the same effect on astrocytosis.

Despite the moderate changes to the behaviour of TAU58/2 mice when B-cells were depleted throughout development and post-natal life, both genetic and antibody-mediated B-cell depletion resulted in a significant reduction of astrocytosis in the amygdala, where GFAP labelling is normally sparse in wild-type mice and markedly increased in TAU58/2 brains together with early NFT pathology^17,26^. Absence of similar changes to astrocytes in the hippocampus may be due to the marked GFAP labelling of this brain area in naïve mice, likely masking effects of B-cell depletion. Given the absence of overt changes to tau pathology in the amygdala in either TAU58/2.muMT^−/−^ or anti-CD20 antibody-treated TAU58/2 mice, our data suggest that the pathways that lead to astrocytosis in TAU58/2 mice involve, at least in part, antibody-mediated processes. Astrocytes have been found to adopt distinct states that can either promote or limit neurodegeneration^22,25,28–31^. To this end, staining of A1 and A2 subpopulations suggests that effects of autoantibodies are astrocyte fate-independent in TAU58/2 brains, but augmented learning deficits in TAU58/2.muMT^−/−^ mice as compared with TAU58/2.muMT^+/+^ littermates together with reduced astrocytosis may suggest that antibody-mediated processes support neuroprotective states of astrocytes during onset.

Autoantibodies to pathologically phosphorylated tau have been reported in humans and tau transgenic mouse models. Their role in disease, however, is unclear. Autoantibody titres against tau were very low, which is very different from auto-immune disease^22,29–31^. Furthermore, it is known that only a very small proportion of circulating antibodies can pass the blood brain barrier (BBB)^32,33^. Therefore, one may conclude that a major disease-driving role of autoantibodies detected in association with tau pathology is rather unlikely. Nevertheless, specific staining of IgG in perfused brain tissue of TAU58/2 mice that was absent in crosses with the muMT^−/−^ line, demonstrate that antibodies do pass the BBB and tend to label areas with more abundant tau hyperphosphorylation, such as the hippocampus. We and others have previously shown that both active and passive immunization of tau transgenic mice limits tau pathology when treatment is initiated before the onset of overt tau pathology^19,34,35^ or halts progression of tau pathology when vaccination commenced after the onset of neuropathological changes, including during late stages of the disease^18,19,36–38^. These studies demonstrated that antibodies specific for distinct phosphorylated tau species can limit tau pathology and hold great promise for therapy development. Here, our finding of unchanged tau histopathology, particularly of the late phosphorylation sites PHF1 and pS422, but changes to tau solubility, suggest that the presence of low titre autoantibodies to pathological tau are not sufficient to limit the progressive tau hyperphosphorylation yet are sufficient to impact on tau aggregation. To achieve therapeutic effects of tau-specific antibodies, much higher titres are required, such as those found during active and passive vaccination trials in different tau transgenic mouse models^18,19,38^. The mechanisms underlying brain area-specific differences in tau solubility upon B-cell depletion and their possible contribution to the functional changes of TAU58.2 mice remain to be investigated.

Taken together, our data suggest that the adaptive immune system (i.e. B-cells and antibodies) do not play a major role in the onset and progression of tau pathology and associated functional deficits in tau transgenic mice. Autoantibodies to pathological tau may however have some subtle disease modulating effects, such as altering learning.

## Methods

### Mice

TAU58/2 mice express the human 0N4R tau isoform with the P301S mutation under the control of the mouse Thy1.2 promoter C57BL6 background^17^. muMT^−/−^ (B6.129S2-Ighm^tm1Cgn^/J, JAX stock #002288) mice have a targeted disruption of a membrane exon of the gene encoding the µ-chain constant region, arresting the development of B lymphocytes at the pre-B lymphocyte stage^23^. Both, female and male mice were used at even numbers. Mice were housed in 12 h/12 h light–dark cycle with food ad libitum (Rodent chow; Gordon Specialty Feeds). All animal experiments were approved by the Animal Ethics Committees of Macquarie University and the University of New South Wales. All procedures complied with the statement on animal experimentation issued by the National Health and Medical Research Council of Australia.

### B lymphocyte depletion

Three-week-old male TAU58/2 mice were injected intravenously fortnightly through the tail vein with 10 mg/kg mouse anti-CD20 antibody (clone5D2, isotype IgG2a) or mouse isotype control antibody (anti-ragweed IgG2a). Both antibodies were kindly provided by Genentech. Animals were humanely killed and tissue harvested at eight months of age. Treatment schedule and effective dose have previously been determined^39^.

### Behavioural, memory and motor testing

The following behavioral tests all used the same cohorts of mice. The CD20-treated cohort consisted of heterozygous TAU58/2^tg/wt^ mice (CD20 n = 12, control n = 12) and their wildtype littermates (CD20 n = 13, control n = 12). Rotarod and elevated plus maze was performed at 3.5 months of age and Morris Water Maze at 7 months of age. muMT^−/−^ mice were crossed with TAU58/2 transgenic mice to obtain a cohort composed of TAU58/2^tg/wt^ × muMT^−/−^ (n = 12), TAU58/2^tg/wt^ × muMT^+/+^ (n = 8), TAU58/2^wt/wt^ × muMT^−/−^ (n = 13) and TAU58/2^wt/wt^ × muMT^+/+^ (n = 13) mice. Rotarod and elevated plus maze were performed at 4 months of age and Morris Water Maze at 8 months of age. The experimenter was blinded to genotype and/or treatment whilst conducting the behavioural tests.

#### Elevated Plus Maze

Anxiety/disinhibition in the mice was assessed as previously described^40^. Briefly, the Elevated Plus maze (Stoelting Co.) consisted of two open and two closed arms (each 35 cm × 5.5 cm), as well as a central platform (5.5 cm × 5.5 cm), elevated 60 cm above the ground. Mice were acclimatized to the room for 1 h prior to testing, then placed on the center platform facing an open arm and recorded for 5 min from above. Videos were analysed using the AnyMaze software.

#### Rotarod

Motor performance of mice was determined using a Rotarod (Ugo Basile) in acceleration mode (5–60 rpm) over 120 s for three consecutive days, as previously reported^17^. The longest time each mouse remained on the rotating wheel out of 5 attempts per session was recorded for each day.

#### Morris water maze

Assessment of spatial learning and memory was performed as previously reported^41^. Briefly, after mice were acclimatized to the procedure rooms (1 h prior to experiments each day) they were placed into a 1.2 m diameter tank filled with opaque water and a submerged invisible 10 cm diameter escape platform opposite the entry quadrant. The tank was surrounded by 4 different cues displayed for each quadrant. During four subsequent trials each day, all mice were given 60 s to locate the escape platform. Only mice that failed to find the platform were thereafter guided to the platform. All mice remained on the platform for 60 s after reaching it and before being returned to their home cage. Starting positions were varied between trial within the quadrant opposite the platform. These acquisition trials were carried out for 6 and 7 consecutive days for muMT^−/−^ crossed and CD20-treated mice, respectively, until control mice reached the criterion of less than 20 s for trial completion. On the following day, the platform was removed, and mice placed into the tank for 30 s to explore (= probe trial). One day later, the platform was equipped with a visible flag, and cues were removed from around the tank, to ensure that all mice had normal vision. Videos were analyzed using the AnyMaze tracking software (Stoelting).

### Flow Cytometry

Following perfusion, spleens were harvested and homogenised into a single cell suspension in phosphate buffered saline (PBS) using a 100 µm Falcon cell-strainer. Red blood cells were lysed using BD FACS Lysing Solution (cat#349202) and the remaining cells blocked with FACS buffer (phosphate buffered saline with 2% fetal bovine serum) and stained using fluorochrome-conjugated antibodies against IgM (IgM-APC, cat#550676, BD Biosciences) and CD45R/B220 (CD45R/B220-PE, cat#553090, BD Biosciences). Cells were run on a BD LSRFortessa SORP (BD Biosciences) flow cytometer and the data analysed with Flow Jo Version 10 software (FlowJo, LLC/Treestar). n = 6 for each genotype and treatment. Representative results are shown in figures.

### Histology

Mice were anesthetized and transcardially perfused with cold phosphate buffered saline. Brains were removed and one hemisphere was immersion-fixed in 4% paraformaldehyde for immunohistochemical analysis. Fixed brains were processed using an automated tissue system (Excelsior, Thermo, USA), embedded in paraffin and coronally sectioned at the level of the mid-hippocampus at 3 µm using a microtome (Thermo, USA) for immunofluorescence staining. Microscopy was performed with an Olympus BX51 (Olympus, USA) epi-fluorescence microscope equipped with an Olympus DP70 software colour camera for representative images. Slides were scanned with an AxioScan (Zeiss) for entire brain region counts using ImageJ software (Fiji). All staining was done in Sequenza staining racks for standardization using previously reported protocols^42^. Following 1 h in blocking buffer (3% (v/v) goat serum (Sigma), 2% (w/v) bovine serum albumin (Sigma) in phosphate buffered saline), slides were incubated with primary antibody in blocking buffer at 4 °C overnight. The following primary antibodies were used: Tau13 (sc-21796, Santa Cruz), PHF-1 (pS396/pS404 tau, P. Davies, Albert Einstein College of Medicine, New York, NY), pS422 tau (ab71495, Abcam), pS214 tau (ab170892, Abcam), GFAP (G9269 Sigma), IBA-1 (019-19741, Wako), C3 (ab11862, abcam) and S100A10 (PA5-95505, Invitrogen). Alexa Fluor secondary antibodies (Invitrogen) were used for detection and counterstaining was done with DAPI (Molecular Probes).

For staining of endogenous IgG in the brain, TAU58/2.muMT^−/−^ and TAU58/2.muMT^+/+^ mice were perfused as above and after fixation were sectioned at 50 μm using a vibratome (Campden, UK). Following permeabilization with 0.05% NP-40 for 10 min and treatment with 3% hydrogen peroxide for 1 h, sections were blocked for 1 h with blocking buffer and then incubated with anti-mouse IgG biotin-coupled secondary antibodies (Sigma) in blocking buffer. The ABC-HRP detection kit (Vector) using ImmPACT DAB (Vector) was used to visualise staining prior to mounting with DPX Mountant for histology (Sigma).

### Sequential tissue extraction

Sequential extraction of brain tissue from different brain areas with buffers of increasing stringency was carried out as previously described in detail by us^19^.

### Western blotting

Western blotting was carried out using established protocols^43^. Primary antibodies were against Tau13 (sc-21796, Santa Cruz), PHF-1 (pS396/pS404 tau, P. Davies, Albert Einstein College of Medicine, New York, NY), pS422 tau (ab71495, Abcam), pS214 tau (ab170892, Abcam) and Gapdh (MAB374, Merck-Millipore). All primary antibodies were visualized with HRP-coupled species-specific secondary antibodies (Santa Cruz) and an HRP substrate (Biorad), using a Chemidoc CCD-camera detector system (Biorad). Quantification of blots was done with Image J FIJI (NIH) and band intensities were normalized to Gapdh band intensities, and tau phosphorylation signals further normalized for human tau levels per fraction. Full size blots presented in Figs. 5 and 10 are shown in Supplementary Fig. 4 and 5.

### Statistical analysis

All statistical analysis was done using the Graphpad Prism 8.0 software (GraphPad, USA) using either Student’s t-tests for comparison of two data sets, analysis of variance (ANOVA) for comparison of more than two data sets or two-way ANOVA for comparison across time. N-numbers are provided in each figure. P < 0.05 values were considered significant. All values are presented as mean ± standard error of the mean.

## Supplementary information

Supplementary information
