# Supplementary material for: Contribution of endogenous antibodies to learning deficits and astrocytosis in human P301S mutant tau transgenic mice
Source: Sci Rep. 2020 Aug 14;10:13845. doi: 10.1038/s41598-020-70845-x (PMC7428012; doi:10.1038/s41598-020-70845-x)
Supplement: Supplementary file 1 — Supplementary information [file 41598_2020_70845_MOESM1_ESM.pdf]

Supplementary material

for

Contribution of endogenous antibodies to learning deficits and astrocytosis in human P301S mutant tau transgenic mice

Julia van der Hoven, Annika van Hummel, Magdalena Przybyla, Prita R Asih, Mehul Gajwani, Astrid F Feiten, Yazi D Ke, Arne Ittner, Janet van Eersel, and Lars M Ittner

Supplementary Figures 1 – 5

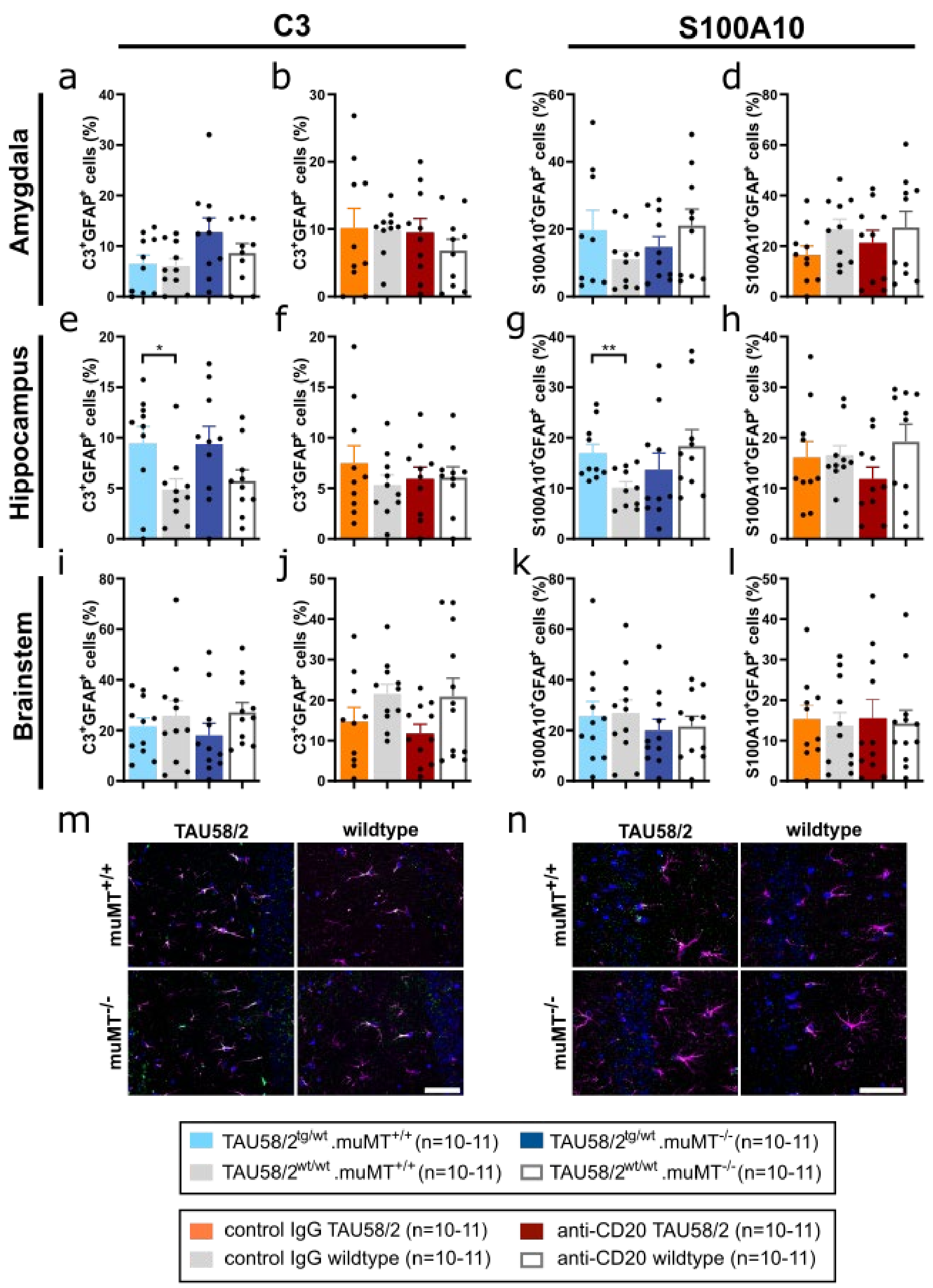

Supplementary Figures 1. Unchanged astrocyte subpopulations in TAU58/2.muMT$^{-/-}$ mice and upon antibody-mediated B-cell depletion in TAU58/2 mice. (**a-l**) Quantification of A1 and A2 astrocyte subpopulations in the (a-d) amygdala, (e-h) hippocampus and (i-l) brainstem by co-staining brains sections with antibodies to (a,b,e,f,i,j) C3 (=A1) and (c,d,g,h,k,l) S100A10 (=A2) with GFAP (=all astrocytes) (*P=0.0328, **P=0.0055). (**m**) Representative co-staining TAU58/2$^{tg/wt}$.muMT$^{-/-}$ brains and TAU58/2$^{tg/wt}$.muMT$^{+/+}$, muMT$^{-/-}$ and muMT$^{+/+}$ controls with antibodies to C3 (green) and GFAP (magenta) and DAPI (blue) for nuclei labelling. (**n**) Representative co-staining TAU58/2$^{tg/wt}$.muMT$^{-/-}$ brains and TAU58/2$^{tg/wt}$.muMT$^{+/+}$, muMT$^{-/-}$ and muMT$^{+/+}$ controls with antibodies to S100A10 (green) and GFAP (magenta) and DAPI (blue) for nuclei labelling. Scale bar = 100µm for all images.

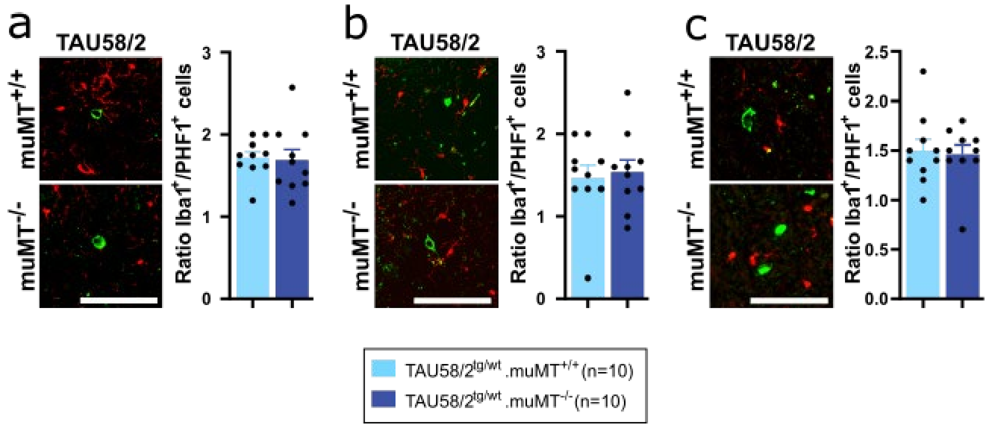

Supplementary Figures 2. Unchanged microglia behaviour in TAU58/2.muMT$^{-/-}$ mice. (**a-c**) Numbers of Iba1^{+} microglia (red) within 50µm radius of tau pathology-harbouring (PHF1^{+}) cells (green) were comparable in TAU58/2$^{tg/wt}$.muMT$^{-/-}$ and TAU58/2$^{tg/wt}$.muMT$^{+/+}$ (a) amygdala, (b) hippocampus and (c) brainstem. Scale bar = 100µm for all images.

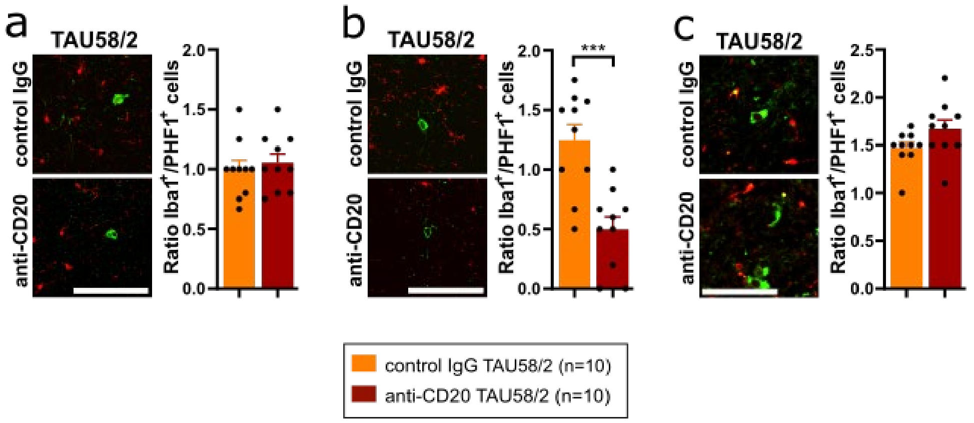

Supplementary Figures 3. Microglia behaviour upon antibody-mediated B-cell depletion in TAU58/2 mice. (**a-c**) Numbers of Iba1^{+} microglia (red) within 50µm radius of tau pathology-harbouring (PHF1^{+}) cells (green) were comparable in (a) amygdala and (c) brainstem but significantly reduced in (b) hippocampus of anti-CD20-treated TAU58/2 mice compared with control IgG-treated TAU58/2 littermates (***P=0.0004).

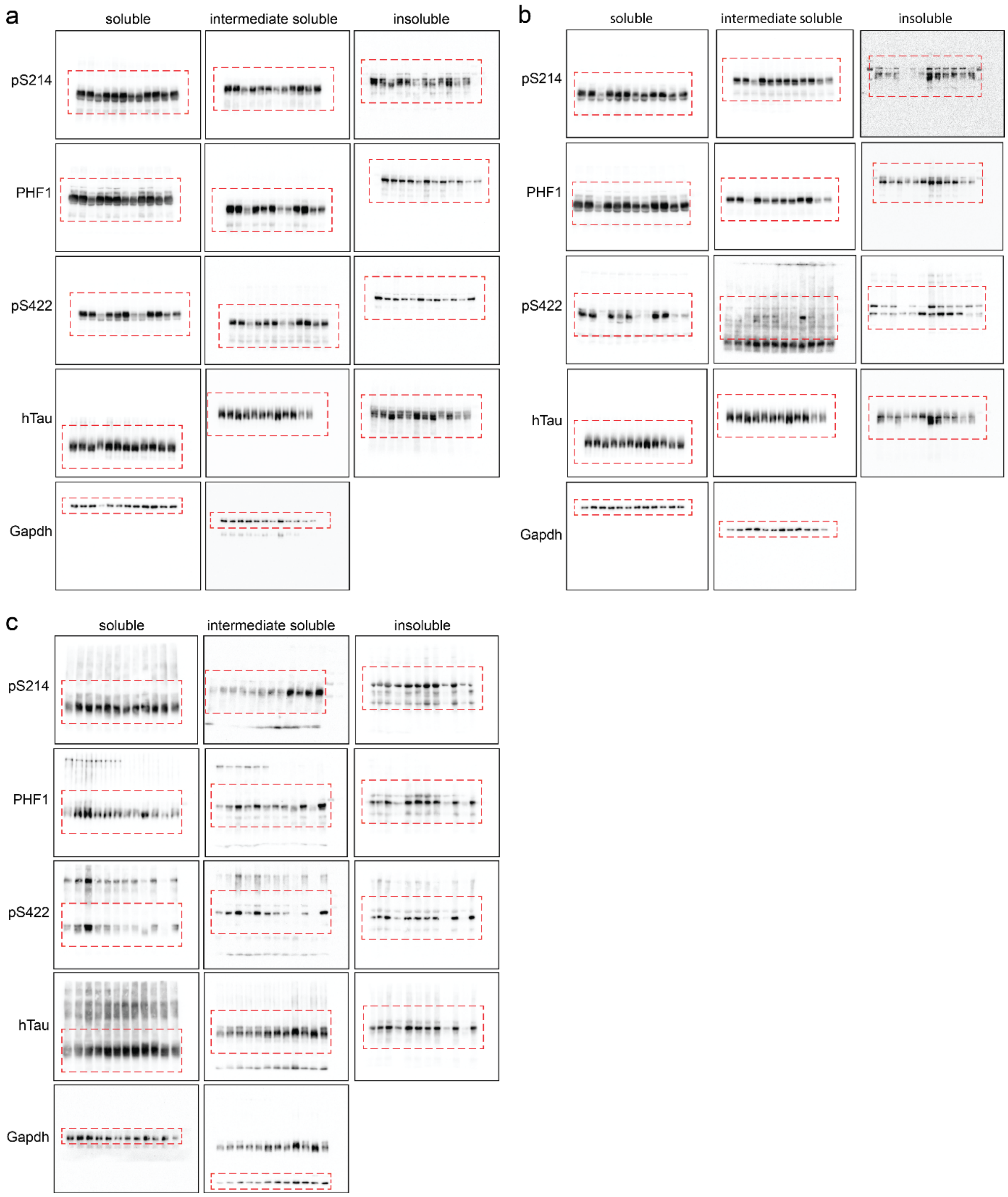

Supplementary Figures 4. Full size blots shown in main Fig. 5. Red broken boxes indicate areas presented in (**a**) Fig. 5a, (**b**) Fig. 5c and (**c**) Fig. 5e.

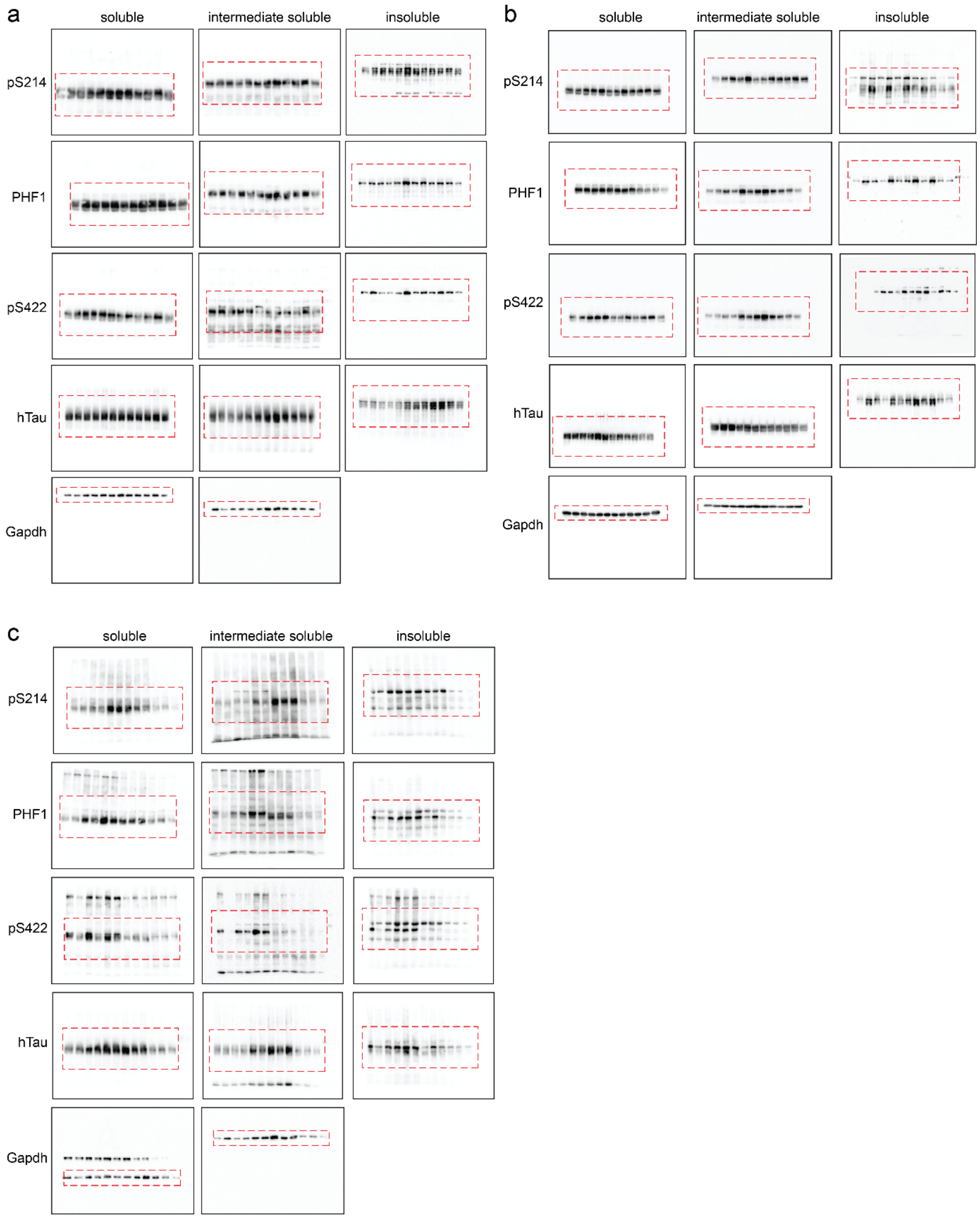

Supplementary Figures 5. Full size blots shown in main Fig. 10. Red broken boxes indicate areas presented in (**a**) Fig. 10a, (**b**) Fig. 10c and (**c**) Fig. 10e.
